# Predicting outcome of rethoracotomy for suspected pericardial tamponade following cardio-thoracic surgery in the intensive care unit

**DOI:** 10.1186/1749-8090-6-79

**Published:** 2011-05-30

**Authors:** Birkitt L ten Tusscher, Johan AB Groeneveld, Otto Kamp, Evert K Jansen, Albertus Beishuizen, Armand RJ Girbes

**Affiliations:** 1 Department of Intensive Care, VU University Medical Center, De Boelelaan 1117, 1081 HV Amsterdam, The Netherlands; 2 Department of Cardiology, VU University Medical Center, De Boelelaan 1117, 1081 HV Amsterdam, The Netherlands; 3 Department of Cardiothoracic surgery, VU University Medical Center, De Boelelaan 1117, 1081 HV Amsterdam, The Netherlands

**Keywords:** regional vs circumferential tamponade, echocardiography, haemodynamics of tamponade, fluid balance, haemodynamic monitoring

## Abstract

**Objectives:**

Pericardial tamponade after cardiac surgery is difficult to diagnose, thereby rendering timing of rethoracotomy hard. We aimed at identifying factors predicting the outcome of surgery for suspected tamponade after cardio-thoracic surgery, in the intensive care unit (ICU).

**Methods:**

Twenty-one consecutive patients undergoing rethoracotomy for suspected pericardial tamponade in the ICU, admitted after primary cardio-thoracic surgery, were identified for this retrospective study. We compared patients with or without a decrease in severe haemodynamic compromise after rethoracotomy, according to the cardiovascular component of the sequential organ failure assessment (SOFA) score.

**Results:**

A favourable haemodynamic response to rethoracotomy was observed in 11 (52%) of patients and characterized by an increase in cardiac output, and less fluid and norepinephrine requirements. Prior to surgery, the absence of treatment by heparin, a minimum cardiac index < 1.0 L/min/m^2 ^and a positive fluid balance (> 4,683 mL) were predictive of a beneficial haemodynamic response. During surgery, the evacuation of clots and > 500 mL of pericardial fluid was associated with a beneficial haemodynamic response. Echocardiographic parameters were of limited help in predicting the postoperative course, even though 9 of 13 pericardial clots found at surgery were detected preoperatively.

**Conclusion:**

Clots and fluids in the pericardial space causing regional tamponade and responding to surgical evacuation after primary cardio-thoracic surgery, are difficult to diagnose preoperatively, by clinical, haemodynamic and even echocardiographic evaluation in the ICU. Only absence of heparin treatment, a large positive fluid balance and low cardiac index predicted a favourable haemodynamic response to rethoracotomy. These data might help in deciding and timing of reinterventions after primary cardio-thoracic surgery.

## Background

Whereas pericardial effusion is relatively common and may not require drainage, pericardial tamponade is a rare but potentially life-threatening complication after cardio-thoracic surgery and opening of the pericardium [1-11]. Recognition is difficult or late because tamponade is often regional rather than circumferential, contributing to relatively non-classical and non-specific findings [3-5,9,11-14]. Regional tamponade is often caused by a blood clot or haematoma with localised effusion and may even surpass detection on echocardiography [4,5,8,9,13,14]. Anticoagulant therapy may be a risk factor, perhaps by promoting intrapericardial haemorrhage [2,6,7,9,15].

Many small series that address the diagnostic problems of pericardial tamponade after cardiac surgery do not incorporate haemodynamic variables as obtained during monitoring in the intensive care unit (ICU) [5-9,11,13]. The latter may facilitate detection of haemodynamic compromise, but data may be confounded by cardiac function, concomitant mechanical ventilation and vasopressor/inotropic therapy. Many patients in whom pericardial tamponade is suspected are, even if delayed, ultimately subjected to rethoracotomy and the haemodynamic response to this treatment can be considered as the reference for a correct diagnosis. The predictors, if any, of a favourable response to rethoracotomy are largely unknown and could possibly help to decide on timing for repeated surgery in patients suspected of pericardial (regional) tamponade after primary cardiac surgery.

The aim of the current study was therefore to evaluate, retrospectively, the clinical, haemodynamic and echocardiographic features that may predict a favourable haemodynamic response to rethoracotomy for suspected pericardial tamponade after recent cardio-thoracic surgery, in a consecutive series of patients in the ICU.

## Patients and methods

### Patients

We included only patients who were in the ICU at the time of diagnosing suspected pericardial tamponade necessitating rethoracotomy, after having undergone primary cardio-thoracic surgery (at maximum 3 weeks earlier), in the period from November 2003 through May 2009 at our institution. In this period 3743 patients underwent cardiac surgery, 259 patients underwent a rethoracotomy (6.9%), mainly for chest tube bleeding. Patients were selected from a registry of cardiac surgery patients, surgical and ICU records. These electronic databases were screened for rethoracotomy and tamponade, individual case were reviewed for inclusion. Exclusion criteria were rethoracotomy for postoperative bleeding alone, after other than cardio-thoracic surgery.

### Data collection

The selection of collected data was based on previously suggested risk factors, clinical signs and echocardiographic features of pericardial tamponade after primary cardiac or aortic root surgery [2]. Electronic patient charts were reviewed to obtain age, sex, weight, Euroscore, previous history of chronic renal insufficiency and use of heparin, acetylsalicylic acid and clopidrogel. The type of primary surgery was retrieved. The chart of the rethoracotomy was evaluated for evacuation of clots and pericardial fluid. In 19 patients echocardiography (Philips Sonos 7500, Philips IE33 and GE Vivid 7) was performed prior to rethoracotomy, and 17 were made transoesophageally and reporting was restricted to the latter. Of these, 14 were available for later reassessment (OK), after blinding to study results. We evaluated the presence of the following features of cardiac tamponade: right atrial collapse, right ventricular collapse, left atrial collapse, left ventricular collapse, increased respiratory variation of mitral blood flow velocities, pericardial effusions, magnitude and location, and identifiable clots [1,4-6,8,11-14]. We used electronic patient charts for collection of haemodynamic parameters including, for worst values within 24 h prior to rethoracotomy, worst value within and at 24 h after rethoracotomy, and for those directly prior to and after rethoracotomy of, heart rate and rhythm, mean arterial pressure (MAP), pulmonary artery occlusion pressure (PAOP), central venous pressure (CVP), cardiac index (CI), mixed or central venous O_2 _saturation (S_v_O_2_), diuresis (mL/h) and fluid balance (mL) per 24 h. We also collected doses (in mg/h per infusion pump) of vasopressor/inotropes used for treatment and selected laboratory parameters such as coagulation times, platelet counts and serum creatinine values, that are assessed daily on routine basis in our unit. We calculated the cardiovascular (CV) component of the Sequential Organ Failure Assessment (SOFA) score, within 24 h before and at 24 h after rethoracotomy, to judge haemodynamic compromise and its improvement upon reintervention. The SOFA score evaluates organ function over time [16] and we assessed the CV component of the score considering this most relevant for our study goal. The CV component of the SOFA score takes MAP and the doses per kg of vasopressor/inotropic therapy used in the treatment of hypotension into account, and ranges from 0 to 4 with 0 indicating normo-tension without treatment. We thus separated patients with and without a decrease of CV SOFA score > 1 within 24 h after rethoracotomy and studied possible predictors of this favourable haemodynamic response to surgery.

Patients otherwise received protocolized standard care in our unit, with pressure-controlled mechanical ventilation and positive end-expiratory pressure (PEEP) and inspiratory O_2 _fraction (FIO_2_) dosed on the basis of arterial PO_2_. Respiratory rate was adjusted to maintain normocarbia while tidal volume was aimed not to exceed 8 mL/kg, recruitment procedures were performed routinely. Haemodynamic monitoring was routinely done with help of a catheter in the radial artery and either a central venous catheter and/or a pulmonary artery catheter (n = 14). The latter allowed to measure the PAOP after proper wedging, the cardiac output and the mixed venous S_v_O_2 _(Radiometer, Copenhagen, Denmark). Pressures were measured at the end of expiration after calibration and zeroing to atmospheric pressure, with patients in supine position. For cardiac output measurements, the bolus thermodilution method was used with help of central venous, room-temperature D5W injections. Triplicate measurements, routinely done after major clinical or therapeutic changes and otherwise once per shift, were averaged (Maquette, Milwaukee, Wisc., USA) and normalized to body surface area calculated from height and weight. Attending physicians gave fluids and vasopressor/inotropic treatments on the basis of severity of haemodynamic compromise and expected haemodynamic responses to such treatments, while awaiting results of diagnostic measures and surgical interventions. For vasopressor therapy, norepinephrine is the drug of first choice in our institution. Mortality refers to death in the hospital.

### Statistical analysis

Data are summarized by median (range) and non-parametric tests were used to compare groups according to the course of CV SOFA after rethoracotomy, including the Wilcoxon signed rank test for paired and the Mann-Whitney U test for unpaired data, because of the small numbers, even though most data were normally distributed (Kolmogorov-Smirnov test P > 0.05). Fisher's exact test was used to compare proportions. Receiver operating characteristic (ROC) curve analysis of sensitivity versus 1-specificity was done for variables different between outcome groups at the P < 0.10 level, yielding an area under the curve (AUC) and cut-off value with highest specificity and sensitivity, to evaluate predictive values of variables for a fall in CV SOFA after rethoracotomy. The κ statistic was used to evaluate reproducibility of the echocardiograms, with respect to number of visible features of potential tamponade. Exact P values are given and considered statistically significant if < 0.05.

## Results

### Clinical features

We identified 21 consecutive patients in the ICU in whom a rethoracotomy was performed because of suspected pericardial tamponade, 1 to 16 (median 3) days after primary cardio-thoracic surgery (Table 1). There were 2 patients with a previous rethoracotomy because of surgical bleeding between primary surgery and rethoracotomy for suspected tamponade. Two patients started renal replacement therapy before rethoracotomy for suspected tamponade, one patient was already on renal replacement therapy for chronic renal insufficiency prior to the first surgery. Eight patients had received heparin in therapeutic doses between primary surgery and rethoracotomy. All patients were on mechanical ventilation, whereas one patient had experienced a cardiac arrest prior to rethoracotomy. Mortality in hospital was 3 (30%) in patients with unchanged and 3 (27%) in patients with decreasing CV SOFA score upon rethoracotomy, respectively (P = 1.0).

**Table 1:** Patient characteristics.

Age, year	Sex, m/f	Weight, kg	EuroScore	Type of primary surgery
61	m	69	6	AVRbio
84	f	75	13	Arch
64	m	100	7	CABG, MVP
74	m	82	8	CABG, AVRbio
61*	f	115	7	AVR, MVP
76	m	61	6	CABG, AVRbio
65	m	89	17	AVRbio
59	f	102	6	AVR, MVR
75	f	84	7	CABG, AVR
78	m	70	6	Arch
65	m	79	2	CABG
75	f	63	6	CABG
76	f	85	10	Arch+ascending
83	m	82	7	CABG
68	m	65	5	CABG
71	f	68	8	CABG
74	f	57	8	CABG
71	m	90	6	AVRbio
68	f	65	10	CABG, Bentall
85	m	66	16	CABG
67	f	92	17	MVR

Abbreviations: AVRbio = aortic valve replacement by biological valve, Arch = aortic arch replacement, CABG = coronary artery bypass grafting, MVP = mitral valve plasty, AVR = aortic valve replacement, Arch + ascending = aortic arch and ascending aorta replacement, MVR = mitral valve replacement, Bentall = aortic valve and arch replacement; *dependent on intermittent haemodialysis.

### Haemodynamic parameters

In the 24 h preceding rethoracotomy for suspected pericardial tamponade, 71% of patients had a period of hypotension (MAP < 60 mm Hg), 80% percent an elevated central venous pressure (> 12 mm Hg), 33% (an episode of) atrial fibrillation and 67% tachycardia (heart rate > 100/min). Minimum urine output was low in patients with and without a decrease in CV SOFA score at 24 h after rethoracotomy (median of 7 and 0 mL/h respectively). Table 2 summarizes haemodynamic and laboratory variables in this period. There was no major difference in the severity of haemodynamic compromise between patients in both groups. The PAOP-CVP gradient did not differ either.

**Table 2:** Haemodynamic and laboratory values within 24 h prior to rethoracotomy for suspected pericardial tamponade as predictors of its haemodynamic benefit.

	CV SOFA unchanged n = 10	CV SOFA decreased n = 11	P
**Haemodynamics**			
Maximum heart rate (b/min)	121 (76-200)	107 (90-193)	0.65
Minimum MAP (mmHg)	55 (5-64)	53 (46-67)	0.47
Minimum CI (L/min/m2)	2.1 (1.7-3.0)	1.4 (1.0-2.6)	0.09
Maximum PAOP (mmHg)	23 (15-33)	18 (11-26)	0.28
Maximum CVP (mmHg)	19 (0-30)	19 (7-27)	1.00
Minimum SvO2 (%)	40 (33-62)	53 (46-67)	0.15
Maximum norepinephrine, mg/h	4.0 (0-8)	1.2 (0-6)	0.17
Maximum dopamine, mg/h	0 (0-80)	0 (0-20)	0.92
CV SOFA	4 (3-4)	4 (2-4)	0.51
Minimum diuresis (mL/h)	0 (0-40)	7 (0-47)	0.43
Fluid balance (mL/24 h)	3,355 (1,184-4,863)	4,828 (2,988-11,205)	0.07
**Laboratory**			
PT, INR	1.6 (1.2-1.8)	1.6 (1.3-4.8)	0.56
aPTT, sec	52 (34-38)	41 (35-69)	0.28
Platelets, ×10^9^/L	151 (58-228)	106 (31-161)	0.28
Creatinine, micromol/L	165 (87-407)	121 (77-310)	0.15

Median (range) or number (percentage), where appropriate; CV SOFA = cardiovascular sequential organ failure assessment score, MAP = mean arterial pressure, CI = cardiac index, PAOP = pulmonary artery occlusion pressure, CVP = central venous pressure, S_v_O_2 _= mixed or central venous O_2 _saturation, PT = prothrombin time, aPTT = activated partial thromboplastin time.

### Echocardiographic parameters prior to rethoracotomy

Echocardiography was performed on indication. In two patients echocardiography was not performed prior to rethoracotomy, because of hemodynamical instability and high clinical suspicion of tamponade these patients went straight to the operating room. In the remaining 19 patients echocardiography was performed, 17 were made transoesophageally. In the two patients with only transthoracic echocardiography, one examination showed a clot next to the right ventricle without compression and no other abnormalities, while the other echo showed a clot behind the left atrium with compression but without collapse of the left atrium together with 1 cm of pericardial effusion.

All but one patients who underwent transoesophageal echocardiography prior to rethoracotomy (n = 17) had a pericardial effusion, which was circumferential in 2 patients only. Only 36% had at least one (range 0-3) echographic sign of possible pericardial tamponade on transoesophageal echocardiography, and none predicted the outcome of rethoracotomy (Table 3). Of 13 clots found on rethoracotomy, 9 (69%) had been identified prior to surgery in patients undergoing transoesophageal echocardiography, whereas there were 2 correct negative, 2 false positive and 4 false negative echocardiographic diagnoses. Twelve visible clots on echocardiography were located anterior to the right atrium, ventricle, or both, while 2 were located posterior. At later reassessment of the preoperative echocardiograms, the number of features per patient suggestive for tamponade was 0-2, with 43% showing at least one feature. In the reassessments, 11 of the 11 clots found at surgery were detected, with 3 false positives. The κ statistic between number of echocardiographic features suspected for tamponade at first and second assessment was 0.23 (P = 0.21).

**Table 3:** Echocardiographic findings prior to rethoracotomy for suspected pericardial tamponade.

	CV SOFA unchanged n = 9	CV SOFA decreased n = 8	P
Pericardial effusion (cm)	2.0 (1.0-4.0)	2.0 (0-4.0)	0.91
Clot	6 (67)	5 (63)	1.0
Right atrial collapse	4 (44)	1 (13)	0.29
Left atrial collapse	0	2 (25)	0.21
Right ventricular collapse	1 (11)	0	1.0
Flow variations	4 (44)	0	0.08
Low end-systolic left ventricular volume	4 (67)	4 (50)	0.63

Median (range) or number (percentage), where appropriate. CV SOFA = cardiovascular sequential organ failure assessment score.

### Response to rethoracotomy

Only 52% of patients haemodynamically improved after rethoracotomy as judged from a decrease in CV SOFA score at 24 h after surgery (Table 4). Patients with a fall in CV SOFA at 24 h after rethoracotomy had an increase in minimum CI, less fluid and norepinephrine requirements on the day after surgery as compared to preoperatively, than patients without such fall in CV SOFA.

**Table 4:** Haemodynamic variables at 24 h after rethoracotomy for suspected pericardial tamponade.

	CV SOFA unchanged		CV SOFA decreased		P for groups
	n = 10	**P vs preop**.	n = 11	**P vs preop**.	
**Within 24 h**					
Max HR (b/min)	112 (83-143)	0.21	104 (76-116)	0.19	0.31
Min MAP (mmHg)	63 (40-67)	0.03	63 (49-76)	0.07	0.65
Min CI (L/min/m2)	1.9 (1.0-2.8)	0.61	2.2 (1.7-2.4)	0.07	0.73
Max PAOP (mmHg)	22 (14-27)	0.60	22 (12-33)	0.69	0.90
Max CVP (mmHg)	15 (9-23)	0.09	17 (7-22)	0.31	0.35
Min S_v_O_2 _(%)	58 (47-74)	0.04	64 (51-75)	0.11	0.49
Max nor, (mg/h)	1.6 (0.2-3.6)	0.01	0.2(0-3.0)	0.005	0.02
Max dop (mg/h)	0 (0-24)	0.28	0 (0-16)	1.0	0.81
Min diuresis (mL/h)	6 (0-50)	1.0	20 (0-45)	0.51	0.31
Fluid balance (mL/24 h)	2,978(507-5,167)	0.77	2,159(-910-3,697)	0.003	0.085
**At 24 h**					
HR (b/min)	96 (73-120)		91 (63-108)		0.92
MAP (mmHg)	77 (64-98)		86 (70-99)		0.15
CI (L/min/m^2^)	2.2 (1.9-3.2)		2.3 (1.9-3.7)		0.62
PAOP (mmHg)	16 (14-21)		16 (6-21)		0.62
CVD (mmHg)	12 (5-17)		13 (7-17)		0.35
S_v_O_2 _(%)	70 (47-77)		65 (60-81)		0.95
Nor, (mg/h)	0.8 (0-2.0)		0 (0-1.8)		0.006
Dop (mg/h)	0 (0-8)		0		0.29
CV SOFA	4 (3-4)		0 (0-3)		na

Median (range) or number (percentage), where appropriate; CV SOFA = cardiovascular sequential organ failure assessment score, preop. = preoperatively, max = maximum, min = minimum, HR = heart rate, MAP = mean arterial pressure, CI = cardiac index, PAOP = pulmonary artery occlusion pressure, CVP = central venous pressure, S_v_O_2 _= mixed or central venous O_2 _saturation, nor = norepinephrine, dop = dopamine, na = not applicable. The change in minimum CI (P = 0.024) and fluid balance from 24 h prior to and after rethoracotomy (P = 0.004) differed between groups.

### Predictors of response prior to and during rethoracotomy

Patients having had heparin between primary surgery and rethoracotomy tended to have less clots (P = 0.09) and had less haemodynamic improvement (P = 0.024) upon rethoracotomy for suspected tamponade. In a ROC curve, a positive fluid balance in the 24 h prior to surgery of 4,683 mL or more had 100% specificity and 45% sensitivity for a fall in CV SOFA upon rethoracotomy with an AUC of 0.78 (P = 0.025). A minimum CI < 1.0 L/min/m^2 ^in the 24 h prior to surgery had 50% sensitivity and 100% specificity for a fall in CV SOFA after rethoracotomy (AUC ROC 0.78, P = 0.023).

All patients had clots identified in the pericardial space at rethoracotomy when responding to surgery whereas only 6 of 10 non-responding patients had such clots (P = 0.035). Hence, the specificity of the presence of clots for postoperative haemodynamic improvement was 100% and sensitivity 65%. The volume of pericardial fluid recovered at rethoracotomy (in n = 9 patients) amounted to 500 (350-1000) mL in patients with unchanged CV SOFA and 800 (600-1700) in patients with a decrease in CV SOFA after rethoracotomy (P = 0.09). The AUC for the ROC curve for improvement upon rethoracotomy of > 500 mL of pericardial fluid removed was 0.89 (P = 0.005), with a specificity of 83% and a sensitivity of 100%.

## Discussion

Our study suggests that clinical, haemodynamic and even echocardiographic features are relatively poor predictors of pericardial tamponade responding to surgical reintervention in ICU patients after primary cardio-thoracic surgery. The data may nevertheless help guiding decisions for rethoracotomy.

Pericardial tamponade has been suggested to occur after cardiac surgery in an early and late form, having different etiologies, with regional obstruction more common in the former and circumferential effusion more frequently encountered in the latter [1-9,11,14]. Regional tamponade can be caused by a blood clot or haematoma with localized effusion and often lacks classical symptoms and signs as well as echocardiographic features [4,5,8,9,13,14]. Tamponade caused by circumferential effusion or regional obstruction is difficult to separate and, in this study, we therefore included all patients who underwent rethoracotomy for suspected tamponade after cardio-thoracic surgery within three weeks after surgery and who were still in the intensive care unit (ICU), in order to study predictors of success of rethoracotomy [9]. The amount of pericardial fluid recovered at rethoracotomy was in the same range as found in other post cardio-thoracic tamponade studies and the median duration to rethoracotomy of 3 days was also comparable [4-6,8,9]. Many small series that address the diagnostic problems of pericardial tamponade after cardiac surgery do not incorporate haemodynamic variables as obtained during monitoring in the intensive care unit (ICU) [5,6,8,9,11,13].

When pericardial tamponade was suspected, 52% of our patients had a improvement of the CV SOFA score, with a rise in cardiac output and less norepinephrine and fluid requirements in the first 24 hours after reintervention. Only few variables predicted the postoperative haemodynamic course, such as the amount of fluids infused prior to rethoracotomy in attempts to increase a low cardiac output. The value of cardiac filling pressures in this context was surprisingly low and the absence of equilibration of pressures, for instance may relate to the predominance of regional versus circumferential tamponade in our patients. Fluid therapy is the primary therapeutic step in the medical treatment of pericardial tamponade, but, depending on pericardial pressure, only half of patients may respond by an increase in cardiac output [15]. It can be surmised that severe inflow limitation would preclude such effect. Apparently, the presence of clots and fluids in the pericardium exerting pressure and thereby obstructing cardiac inflow, is hard to predict by clinical and haemodynamic features.

We studied both echocardiographic features as well as haemodynamic variables, since the former may be regarded as superior for tamponade detection. However, the value of echocardiography in predicting a favourable outcome to rethoracotomy was also disappointing in our series. Only a minority (36%) of the patients with suspected tamponade had at least one echographic sign of possible pericardial tamponade on transoesophageal echocardiography, and none predicted the outcome of rethoracotomy. Some clots and fluids found at surgery and associated with haemodynamic improvement after evacuation, were not detected preoperatively by echocardiography. The usefulness of echocardiography may depend in part on the expertise of the examiner. Therefore, echocardiograms were reassessed by a senior cardiologist (OK). However, this reassessment of echocardiograms did not improve its diagnostic value.

It is increasingly suggested that echocardiographic abnormalities may not fully predict haemodynamic sequelae and that, conversely, even small circumferential effusions may compromise haemodynamics [3,5,7,11,13,14]. Indeed, if abnormalities detected by echocardiography are followed by pericardial evacuation and this does not result in haemodynamic relief, the diagnostic value of the technique can be doubted. Hence, the question remains whether and when surgical reintervention is necessary or not, in a critically ill patient with haemodynamic compromise after prior cardio-thoracic surgery. We intended to contribute to such decision making by comparing haemodynamic and echocardiographic findings in patients with or without a decrease in severe haemodynamic compromise, according to the cardiovascular component of the sequential organ failure assessment (SOFA) score, after rethoracotomy for suspected pericardial tamponade.

Suggested risk factors for pericardial tamponade after cardiac surgery diagnosed by more or less classical clinical and echocardiographic features include primary closure of the pericardium, anticoagulation, female gender, valvular surgery and others [2,6,7,9,17]. Anticoagulant therapy may be a risk factor, perhaps by promoting intrapericardial haemorrhage [2,6,7,9,15]. In our study, prior heparinization seemed to protect rather than to increase the risk for pericardial tamponade, as suggested previously. This may be caused, in part, by decreased rather than increased clot formation with less obstruction, in the presence of adequate drainage [10].

The limitations of this retrospective study include the relatively low number of patients, selected on the basis of strict inclusion criteria. In this study we aimed to identify predictors for the effect of rethoracotomy in patients with suspected tamponade. We may not have inadvertedly excluded patients with suspected tamponade not undergoing reintervention, since we do not manage these patients conservatively. Conversely, we cannot decide on the value of rethoracotomies that are not associated with clear haemodynamic improvement in patients with severe haemodynamic compromise after primary cardio-thoracic surgery. Indeed, reduction of norepinephrine requirements in the group without decrease a in CV SOFA may partly relate to less severe tamponade relieved by surgery. This does not invalidate our conclusions, however. We also cannot speculate on the greater contribution of poor preoperative cardiac function and further deterioration upon primary surgery, even though postoperative transmural infarctions were not detected, in the group with unchanged SOFA.

## Conclusion

Clots and fluids in the pericardial space causing regional tamponade and responding to surgical evacuation after primary cardio-thoracic surgery, are difficult to diagnose preoperatively, by clinical, haemodynamic and even echocardiographic variables obtained in the ICU. Our data suggest that in patients with severe haemodynamic compromise in the ICU after primary cardio-thoracic surgery, without heparin but having a marked positive fluid balance and low CI, regional pericardial tamponade by clots and fluids amenable to surgical decompression should be considered.

## List of abbreviations

ICU: intensive care unit; SOFA score: sequential organ failure assessment score; CV: cardiovascular; MAP: mean arterial pressure; PAOP: pulmonary artery occlusion pressure; CVP: central venous pressure; CI: cardiac index; PEEP: positive end expiratory pressure; S_v_O_2_: mixed or central venous O_2 _saturation; ROC: receiver operating characteristics; AUC: area under the curve

## Competing interests

The authors declare that they have no competing interests.

## Authors' contributions

BLT and ABJG wrote most part of this manuscript. OK reassessed all echocardiograms and gave some comments for this manuscript. AB, ARJG and EKK gave some comments on this manuscript. All the authors read and approved the final manuscript.

## References

[B1] Weitzman LB Tinker WP Kronzon I Cohen ML Glassman E Spencer FC The incidence and natural history of pericardial effusion after cardiac surgery -- an echocardiographic study Circulation 1984 69 3 506 511 6692512 10.1161/01.cir.69.3.506

[B2] Ikäheimo MJ Huikun KEJ Korhonen UR Linnaluoto MK Tarkka MR Takkunen JT Pericardial effusion after cardiac surgery: incidence, relation to the type of surgery, antithrombotic therapy, and early coronary bypass graft patency Am Heart J 1988 116 97 102 10.1016/0002-8703(88)90255-4 3260740

[B3] D'Cruz IA Dick A Pai GM Kamath MV Large pericardial effusion after cardiac surgery: role of echocardiography in diagnosis and management South Med J 1989 82 3 287 291 10.1097/00007611-198903000-00003 2922619

[B4] Russo AM OConner WH Waxman HL Atypical presentations and echocardiographic findings in patients with cardiac tamponade occurring early and late after cardiac surgery Chest 1993 104 71 78 10.1378/chest.104.1.71 8325120

[B5] Chuttani K Tischler MD Pandian NG Lee RT Mohanty PK Diagnosis of cardiac tamponade after cardiac surgery: relative value of clinical, echocardiographic, and hemodynamic signs Am Heart J 1994 127 913 918 10.1016/0002-8703(94)90561-4 8154431

[B6] Pepi M Muratori M Barbier P Doria E Arena V Beti M Celeste F Guazzi M Tamborini G Pericardial effusion after cardiac surgery: incidence, site, size and haemodynamic consequences Br Heart J 1994 72 327 331 10.1136/hrt.72.4.327 7833189 PMC1025541

[B7] Ball JB Morrison WL Experience with cardiac tamponade following open heart surgery Heart Vessels 1996 11 39 43 10.1007/BF01744598 9119804

[B8] Bommer WJ Follette D Pollock M Arena F Bognar M Berkoff H Tamponade in patients undergoing cardiac surgery: a clinical-echocardiographic diagnosis Am Heart J 1995 130 1216 23 10.1016/0002-8703(95)90145-0 7484772

[B9] Kuvin JT Harati NA Pandian NG Bojar RM Khabbaz KR Postoperative cardiac tamponade in the modern surgical era Ann Thorac Surg 2002 74 4 1148 1153 10.1016/S0003-4975(02)03837-7 12400760

[B10] Eryilmaz S Emiroglu O Eyileten Z Akar R Yazicioglu L Tasoz R Kaya B Uysalel A Ucanok K Corapcioglu T Ozyurda U Effect of posterior pericardiac drainage on the incidence of pericardial effusion after ascending aortic surgery J Thorac Cardiovasc Surg 2006 132 27 31 10.1016/j.jtcvs.2006.01.049 16798298

[B11] Imren Y Tasoglu I. Oktar GL Benson A Naseem T Cheema F Unal Y The importance of transesophageal echocardiography in diagnosis of pericardial tamponade after cardiac surgery J Card Surg 2008 23 450 453 10.1111/j.1540-8191.2008.00581.x 18462344

[B12] Reichert CLA Visser CA Koolen JJ van den Brink RBA van Wezel HB Meyne NG Dunning AJ Transoesophageal echocardiography in hypotensive patients after cardiac operations J Thorac Cardiovasc Surg 1992 104 321 326 1495293

[B13] Beppu S Tanaka N Nakatani S Ikegami K Kumon K Miyatake K Pericardial clot after open heart surgery: its specific localization and haemodynamics Eur Heart J 1993 14 230 234 8449199 10.1093/eurheartj/14.2.230

[B14] Price S Prout J Jaggar SI Gibson DG Pepper JR Tamponade following cardiac surgery: terminology and echocardiography may both mislead Eur J Cardiothorac Surg 2004 26 6 1156 1160 10.1016/j.ejcts.2004.08.020 15541977

[B15] Malouf JF Alam S Gharzeddine W Stefadouros MA The role of antico-agulation in the development of pericardial effusion and late tamponade after cardiac surgery Eur Heart J 1993 14 1451 1457 8299624 10.1093/eurheartj/14.11.1451

[B16] Ferreira FL Bota DP Bross A Mlot C Vincent JL Serial evaluation of the SOFA score to predict outcome in critically ill patients JAMA 2001 286 1754 1758 10.1001/jama.286.14.1754 11594901

[B17] Sagristà-Sauleda J Angel J Sambola A Permanyer-Miralda G Hemody-namic effects of volume expansion in patients with cardiac tamponade Circulation 2008 117 1545 1549 10.1161/CIRCULATIONAHA.107.737841 18332261

